# A Clinician’s Guide for Trending Cardiovascular Nutritional Controversies in 2026

**DOI:** 10.1016/j.jacadv.2026.102591

**Published:** 2026-02-09

**Authors:** Michael Miller, Monica Aggarwal, Kathleen Allen, Romit Bhattacharya, Lily N. Dastmalchi, Penny M. Kris-Etherton, Elizabeth Klodas, Dariush Mozaffarian, Robert J. Ostfeld, Kristina S. Petersen, Koushik R. Reddy, Emilio Ros, Vall Vinaithirthan, Andrew M. Freeman

**Affiliations:** a Department of Medicine, Corporal Michael J Crescenz VAMC and Hospital of the University of Pennsylvania, Philadelphia, Pennsylvania, USA; b Division of Cardiology, Department of Medicine, University of Florida, Gainesville, Florida, USA; c Department of Medicine, Massachusetts General Hospital, Boston, Massachusetts, USA; d Department of Cardiology, Inova Health Systems, Fairfax, Virginia, USA; e Department of Nutritional Sciences, Pennsylvania State University, University Park, Pennsylvania, USA; f Step One Foods, Minneapolis, Minnesota, USA; g Food is Medicine Institute, Friedman School of Nutrition Science and Policy, Tufts University, Boston, Massachusetts, USA; h Division of Cardiology, Montefiore Health System, Bronx, New York, USA; i Division of Cardiology, James A. Harvey VA Medical Center, University of South Florida, Tampa, Florida, USA; j Institut d'Investigacions Biomediques August Pi Sunyer, Hospital Clinic, Barcelona, Spain; k Department of Medicine, University of Colorado School of Medicine, Aurora, Colorado, USA; l Division of Cardiology, Department of Medicine, National Jewish Health, Denver, Colorado, USA

**Keywords:** beef tallow, full-fat dairy, seafood, seed oils, triglycerides, ultra-processed foods

## Abstract

Although the broad outlines of a healthy diet are clear, controversy has arisen surrounding certain foods and nutrients. This review updates contemporary nutrition controversies and the extent to which they may promote or protect against cardiovascular disease (CVD). In this review, beef tallow, ultraprocessed foods, full-fat dairy, seed oils, medium chain triglyceride oils, seafood, and alternative sweeteners are considered. Three groupings included: 1) evidence of harm with a recommendation to limit or avoid; 2) lacking in evidence for harm or benefit; and 3) evidence of benefit. The evidence of harm category included beef tallow, due to association with increased low-density lipoprotein cholesterol, ultraprocessed foods associated with worsened cardiometabolic health, and artificial sweeteners owing to correlations with increased CVD. Within the category lacking in evidence were full-fat dairy, medium chain triglyceride, monk fruit, and stevia. Finally, evidence of benefit included seed oils and seafood based on improved CVD outcomes.

## Highlights

• Heart-healthy diets remain a cornerstone of cardiovascular disease prevention.
• Some dietary components remain controversial for cardiovascular health and outcomes.
• Evidence-based recommendations are provided for controversial foods and non-nutrient sources.

A diet high in saturated fat has long been implicated in promoting risk of cardiovascular disease (CVD) owing to a variety of factors including higher concentration of low-density lipoprotein cholesterol (LDL-C).^1^ Recently, however, the potential for varying CVD effects of certain saturated fat products (eg, beef tallow, full-fat dairy, and medium chain triglyceride oils) have gained attention. At the same time, public concerns have arisen about potential harms of seed oils, popularized by social media influencers,^2^ based on the ratio of omega-6 to omega-3 fatty acids, chemical contaminants, or potential proinflammatory effects.^3^ These considerations have intersected; for example, several U.S. chain restaurants have replaced seed oils with beef tallow,∗ with others considering phasing out seed oils.∗∗ Yet are there compelling data that implicate seed oils for the deterioration in cardiometabolic health, or that support beef tallow (and/or tropical oils) as a healthier alternative? The 2025 Dietary Guidelines Advisory Committee (DGAC)^4^ could not draw a conclusion about the effect of lower fat dairy compared to whole fat dairy for cardiometabolic outcomes, including blood cholesterol levels, because of limited evidence in children and adults, whereas the Make America Health Again Strategy announced plans to return whole fat dairy to public schools. Does current evidence support this? In addition, as concerns over health effects of added sugar have increased, the use of alternative sweeteners has increased; what are the implications for cardiometabolic health? Finally, although seafood possesses inherent cardioprotective properties, what safeguards can be recommended to minimize exposure to environmental toxins present in selected species? As these topics have not been examined in recent nutrition controversy papers,^5^, ^6^, ^7^ the goal of the current review is to examine the scientific data to inform clinical practice as it relates to contemporary controversial topics in food and nutrition impacting cardiovascular health.

## Beef tallow

### Food description

Beef tallow, the purified form of beef fat that is suitable for cooking, is high in saturated fat (∼50% of total fatty acids, principally palmitic acid).

### Evidence for the product

Proponents of beef tallow suggest that potential benefits may be derived from nutrients present in small amounts such as choline, fat-soluble vitamins, and conjugated linoleic acid. However, clinical evidence demonstrating any net health benefits is lacking.

### Evidence against the product

Several clinical trials have examined the effects of beef tallow/beef fat on LDL-C compared to unsaturated fats and oils. After consuming a high-fat meal containing beef tallow, LDL-C levels increased approximately 9.3%.^8^ Another study of middle-aged men assigned to 3 weeks of a liquid-formula diet consisting of 40% energy intake derived from beef fat or olive oil produced higher increases of LDL-C on beef tallow than olive oil.^9^ In a randomized crossover study of 14 subjects with LDL-C >130 mg/dL assigned to dietary phases that included enrichment in beef tallow and corn oil, the mean LDL-C was higher following beef tallow assignment (140 mg/dL [3.62 mmol/L]) than corn oil (124 mg/dL [3.23 mmol/L]).^10^ Interestingly, a recent randomized clinical trial (RCT) found consumption of monounsaturated fatty acid (MUFA)-enriched Wagyu-Cross beef to be associated with reduced levels of LDL-C compared to commercial beef. However, this finding could have been due to the greater weight loss in the Wagyu-Cross beef group.^11^ In addition to the effects on LDL-C, beef tallow may exhibit other negative cardiometabolic effects. They include endothelial dysfunction when used as high-heat cooking fat,^12^ impaired insulin sensitivity,^13^ promotion of hepatic steatosis,^14^ and reduced microbial diversity.^15^ Taken together, each 5% isocaloric replacement of animal-based saturated fats (eg, as found in beef tallow) for unsaturated fat raises risk of CVD approximately 10%.^16^ In summary, no compelling data support beef tallow as a healthier cooking option than seed oils or other sources of unsaturated fats.

### Dietary guideline recommendations

Since 1980, the Dietary Guidelines for Americans (DGAs) issued by the Department of Health and Human Services and United States Department of Agriculture have included a recommendation to limit saturated fat, with a 2002 National Academies of Medicine Dietary Reference Intake “as low as possible while consuming a nutritionally adequate diet”.

In 2015, the DGAs introduced the message, replace saturated fats with unsaturated fat with emphasis on unsaturated fat, that is, polyunsaturated and monounsaturated fats. In alignment, the 2021 American Heart Association (AHA) Dietary guidance recommends replacing animal fats with liquid nontropical plant oils.^17^ As described subsequently, health effects of saturated fat may also vary depending on the types of saturated fat and the food source.

### Beef tallow: the bottom line

Consistent evidence demonstrates that saturated fat from beef tallow raises LDL-C, a causal risk factor for atherosclerotic CVD, compared to seed oils and other plant-based oils that are liquid at room temperature and higher in unsaturated fats. Collectively, no evidence supports using beef tallow as a healthier alternative to seed oils or other plant-based oils that are solid at room temperature (eg, palm, palm kernel, or coconut oils).

## Ultraprocessed foods

### Food description

Ultraprocessed foods (UPFs) are industrially prepared products typically made from extracted ingredients that contain artificial and other industrial additives, with minimal, if any, whole food components.^18^ UPFs are typically high in saturated fats, added sugars, and sodium, have excessive caloric content, and have usually low nutrient density.^19^ Examples include sugar-sweetened beverages (SSBs) and many breakfast cereals and energy bars, packaged snacks, reconstituted meat products, and ready-to-heat meals. These products are designed to be inexpensive, convenient, and shelf stable; yet, they commonly fail to provide healthy nutritional value and may pose health concerns. UPF consumption accounts for more than half of calories consumed by Americans.^20^

### Evidence for the product

Current evidence shows no CVD benefit to consuming UPF and there are no demonstrable favorable CVD outcomes related to its use.

### Evidence against the product

In more than 80 prospective studies, higher UPF intake is linked to increased risk of various health conditions, including obesity, hypertension, diabetes, CVD, certain cancers, and premature death.^21^ Three small RCTs of UPF have evaluated the effects on caloric intake and body weight. UPF intake leads to increased energy consumption, independent of fat, protein, carbohydrate, sugar, fiber, or salt content; and higher body weight.^22^, ^23^, ^24^ Although it is clear that higher UPF consumption, on average, worsens health, there can be significant variation in the health implications of various UPF. For example, an otherwise healthful yogurt or whole grain product that has 1 industrial additive may be defined as UPF but still possess health benefits.^25^^,^^26^ Categories of UPF most linked to health harms include sugary beverages, processed meats, and ready-to-heat or ready-to-eat meals. Categories linked in some studies to health benefits include UPF yogurts and whole grains.

### Considerations for dietary recommendations around UPF

#### Nutritional imbalance

UPFs are especially problematic when high in refined grains (most commonly refined wheat, rice, or corn flour or starch), added sugars, and sodium, whereas low in dietary fiber and important micronutrients. Healthier grain products generally have a carbohydrate:fiber ratio of 10:1 or less (at least 1 g of fiber for every 10 g of carbohydrate) and a total fat to saturated fat ratio of 3:1 or more.

#### Loss of natural food structure

In contrast to UPF, whole or minimally processed foods have at least partly intact cellular structure, for instance, the intact physical relationship between the bran, germ, and starch in a whole grain. In contrast, destruction of the food matrix in UPF can affect intestinal transit time, digestibility, and bioavailability of ingested nutrients—impacting satiety, microbiome function, and GLP-1 production.^27^ This likely contributes to the higher energy intakes seen when human subjects are offered UPF rather than whole foods in ad-libitum feeding trials.

#### Additives

Various additives in UPF, such as artificial sweeteners and emulsifiers, may disrupt gut microbiota^28^ and metabolic processes. Others, such as artificial colors, have been linked to the development of cancer and behavioral disorders. Although these compounds are currently allowed for use by the Food and Drug Administration (FDA), many of them are banned in Europe, Canada, Australia, and other countries; and more than 40 U S. states have recently proposed or passed bans on certain chemical food additives. Growing evidence suggests potential harms of several of these additives, as well as potential “cocktail” effects of multiadditive mixtures that cause harm.^29^, ^30^, ^31^

#### Contaminants

The packaging materials and long shelf life of UPF often result in migration of contaminants, such as phthalates, bisphenols, mineral oils, and microplastics from contact packaging. These may increase the risk of cancer, CVD, obesity, insulin resistance, and diabetes.^32^

#### Overconsumption

The hyperpalatable nature of UPF can lead to increased energy intake, contributing to weight gain and metabolic disorders.

### Dietary guideline recommendations

The 2025 DGAC report highlighted the potential risk of obesity with a dietary pattern high in UPF^4^ whereas the 2021 AHA Dietary Guidance statement explicitly recommended to “choose minimally processed foods instead of UPFs”.^17^ This may be best accomplished by reducing specific classes of nutrients and products of concern (eg, sodium, sugar/refined carbs, processed meats).

### UPF: the bottom line

UPF are ubiquitous in the American diet, with substantial evidence linking them to adverse cardiometabolic and other negative health outcomes. Patients should be counseled to limit these UPFs while increasing intake of whole or minimally processed foods.

## Full-fat dairy

### Food description

Milk and dairy products, a staple for most humans, are complex foods, providing macronutrients such as carbohydrates (5 g per 100 mL of milk), protein and fat (about 3 g each), essential minerals such as potassium, phosphorus, magnesium, and calcium, and micronutrients such as vitamin D. Milk fat is made up of characteristic odd-chain saturated fatty acids, pentadecanoic (C15:0) and heptadecanoic (C17:0) acids. Compared to low-fat dairy, the impact of full-fat dairy products, including whole milk, yogurt, and cheese, on cardiometabolic health remains controversial.

### Evidence for the product

The 2025 DGAC committee^4^ found that there was insufficient evidence to draw a conclusion about the effect of consuming higher-fat dairy compared to lower-fat dairy on blood lipid levels, blood pressure and body weight in adults and children, and CVD death in adults. Furthermore, the committee concluded that evidence rated as “limited” suggests that substituting higher-fat dairy with lower-fat dairy is not associated with a difference in the risk of CVD morbidity in adults. A dose-response meta-analysis of 29 prospective cohorts did not identify positive associations between full-fat dairy and CVD or total mortality,^33^ whereas other meta-analyses suggest potential benefits of dairy fat consumption for incident diabetes.^34^ A pooled individual-level analysis of 16 prospective cohorts found higher levels of objective blood biomarkers of dairy fat associated with lower incidence of diabetes.^35^ When individual dairy foods are considered, cheese—a major source of dairy fat—is generally associated with neutral or lower risk of stroke and diabetes.^36^ A Mendelian randomization of 182,000 individuals from 18 studies found dairy intake to be associated with increased lean body mass, consistent with smaller controlled trials on this outcome.^37^

### Evidence against the product

Full-fat dairy products provide meaningful amounts of saturated fat per serving (ie, 5-10 g), thereby contributing to total dietary saturated fat and related increases in LDL-C. In this regard, RCTs have found that substituting low-fat for full-fat dairy reduces LDL-C 5% to 9%.^38^

Prospective observational studies and RCTs have evaluated the risk of CVD (ie, coronary heart disease or stroke) when either full-fat or low-fat milk is compared to red meat. When 5% of energy derived from dairy fat was replaced with the same energy from red meat, the risk of CVD with dairy fat was 6% lower. However, similar energy exchange between dairy and unsaturated fat sources (eg, fish and nuts), results in a 10% to 24% higher CVD risk with dairy fat,^39^ thereby suggesting a hierarchy of dietary-mediated CVD risk starting with fat derived from beef sources, then full-fat dairy, then seafood and plant sources.

### Dietary guideline recommendations

The 2025 DGAC report^4^ and medical societal guidelines recommend limiting the consumption of full-fat dairy. This reduction is recommended primarily because approximately 65% of dairy fat is saturated and replacing saturated fat with unsaturated fats improves LDL-C.^1^ Similarly, the 2021 AHA Scientific Statement^17^ did not recommend full-fat dairy as part of a heart healthy pattern but advised selecting low-fat or fat-free dairy products to limit saturated fat intake and reduce CVD risk. However, some of the data evaluating saturated fat and CVD risk derive from meat products, for which palmitic acid is the predominant saturated and long-chain fatty acid, compared to dairy products that contain significant amounts of medium and short chain saturated fats.^40^

### Full-fat dairy: bottom line

Based on the totality of data, there is insufficient evidence to recommend full-fat dairy over low-fat dairy, or *vice versa*, as part of an overall healthy dietary pattern to minimize cardiometabolic burden and related mortality. Regardless of fat content, dairy products appear superior to red meat sources, although *less* optimal than plant-based protein sources (eg, nuts, legumes, and soy) from a cardiometabolic health standpoint.

## Seed oils

### Food description

Seeds contain numerous beneficial nutrients that sustain the growth of a future seedling, among them healthy fats, vitamins, minerals, protein, and numerous bioactive phytochemicals to protect against a range of environmental stressors. All the major classes of fats in seeds that are used to produce commonly called “seed oils”, such as from canola, soybean, sunflower, and corn, are beneficial for health, including omega-6 polyunsaturated fatty acids (PUFAs) (eg, linoleic acid), omega-3 PUFA (eg, α-linolenic acid), and MUFA (ie, oleic acid) (Table 1). These fats have been studied in numerous controlled trials, with well documented benefits on blood lipids/lipoproteins, glucose, and insulin levels.

**Table 1:** Average Fatty Acid Composition of Selected Common Seed Oils, (Grams Per 100 g)

Fatty Acids	Canola	Soybean	Sunflower	Corn
Saturated	6.6	14.9	9.0	13.4
Monounsaturated	62.6	22.1	63.4	27.7
Polyunsaturated	25.3	57.6	20.7	52.9
Linoleic	17.8	50.9	20.6	51.9
α-Linolenic	7.5	6.6	0.2	1.0

Source: U.S. Department of Agriculture Food Data Central. Accessed September 10, 2025. https://fdc.nal.usda.gov/fdc-app.html#/.

### Evidence for the product

Canola oil and soybean oil are among the most studied seed oils. Each provide a balanced combination of healthy fats, including MUFA and both omega-6 and omega-3 PUFA^5^ (Table 1). The omega-3 content (mainly α-linolenic acid) may be particularly important, as there are not meaningful plant sources of this essential fatty acid.^41^ Health effects of canola oil have been studied in more than 100 RCTs.^42^ Canola oil improves blood cholesterol levels and modestly reduces body weight, especially in the setting of type 2 diabetes or when replacing saturated fat.^43^ In a meta-analysis of 42 trials, canola oil had significantly better effects on blood lipids than other plant oils, including better effects than olive oil on total cholesterol, LDL-C, triglycerides, and the ratio of LDL-C: high-density lipoprotein cholesterol.^44^ The healthy fats in canola oil, especially the omega 6 PUFA, also improve blood glucose, insulin resistance, and insulin production.^45^ Consistent with these benefits and contrary to popular belief, canola oil and sunflower oil also reduce inflammation, with similar effects as olive oil.^46^

RCTs have not found proinflammatory effects of replacing saturated fats with PUFA from seed oils, considering markers like C-reactive protein, interleukins, or tumor necrosis factor-alpha.^47^ In long-term observational studies, higher intake of omega-6 fats associates with lower risk of heart disease, stroke, diabetes, and death from all causes.^48^, ^49^, ^50^, ^51^, ^52^, ^53^ Results from studies using objective blood biomarkers of omega-6 PUFA, such as circulating levels of linoleic acid, which cannot be endogenously synthesized and thus reflect dietary intake, are consistent with dietary data, demonstrating beneficial associations with incident CVD, CVD mortality, and diabetes.^48^, ^49^, ^50^, ^51^ Numerous RCTs have demonstrated favorable effects of omega-6 fats on major cardiometabolic risk factors, including LDL-C, triglycerides, glucose, insulin, hemoglobin A1c, insulin resistance, and insulin sensitivity.^53^ Linoleic acid, the main omega-6 PUFA in seed oils, does not convert efficiently to arachidonic acid, considered a potentially proinflammatory metabolite. Indeed, omega-6 PUFA gives rise to unique metabolites with potent anti-inflammatory effects, such as lipoxins and other specialized proresolving mediators.^52^

### Evidence against the product

Recently, social media and other public outlets have created confusion by suggesting that seed oils are harmful,^54^ despite the lack of clinical trial evidence. This appears to stem from an admixture of only partly related issues, such as the established harms of partially hydrogenated vegetable oils, the harms of UPF, and a concern that omega-6 fats are proinflammatory in humans. Although partially hydrogenated vegetable oils, including partially hydrogenated seed oils, as commonly used in the 1980s and 1990s, were high in industrial *trans* fats, these oils have since been removed from the Generally Recognized as Safe category by the U.S. FDA, and have been largely eliminated from the U.S. food supply.^55^

Unfortunately, seed oils are common in UPF, but these oils are also used widely in home cooking, restaurants, and non-UPF products. Research over the last decade has evaluated the adverse health consequences of UPF,^21^, ^22^, ^23^, ^24^, ^25^, ^26^ but the most relevant drivers appear unrelated to seed oils—which may be among the healthiest components of UPF—and instead, as described previously, to excess starch, sugar, salt, and saturated fat; loss of natural intact food structure, fiber, and phenolics; and artificial chemical additives.

A particular contributor to confusion has been a focus on the omega-6 to omega-3 ratio. Although this ratio is associated with health risks, such associations are driven by the denominator—the amount of omega-3 in the diet—rather than the numerator. Thus, altering the ratio by increasing or decreasing omega-3 levels does influence health, whereas altering the ratio by decreasing omega-6 intake may worsen health.^53^

### Dietary guideline recommendations

Both the 2021 AHA Scientific Statement^17^ and 2025 DGAC report recommend unsaturated fat consumption, including of PUFA, from plant sources. These recommendations include seed oils as preferable to solid fats (eg, butter, lard, and beef tallow).

### Seed oils: bottom line

Evidence from prospective studies and RCTs consistently demonstrate that seed oils provide cardiometabolic benefits, without evidence of proinflammatory concerns in human studies. Seed oils should not be heated to excessive temperatures or subjected to repeated heating (as is often performed in many commercial kitchens).

## Medium-chain triglyceride oils

With the sudden popularity of ketogenic diets on social media has come a renewed discussion of a concept discussed in the medical literature since the 1960s:^56^ medium-chain triglycerides (MCTs). Ketogenic diets and MCTs are linked in the maintenance of ketosis and have certain clinical applications for individuals with specific medical conditions. However, their broad application to the general public, particularly among sedentary individuals, raises significant concerns vis-a-vis lipid profiles, hepatic fat accumulation, and long-term cardiometabolic risk.

### Food description

MCTs are saturated fats with fatty-acyl chains containing 6 to 12 carbon atoms, such as caproic (C6), caprylic (C8), capric (C10), and lauric (C12) acid. Natural sources include coconut oil (∼15% C8–C10, ∼45% C12), palm kernel oil (∼7% C8–C10, ∼48% C12), and full-fat dairy products, which contain primarily C12. As these sources contain saturated fat, the presumptive consequences have been elevated the LDL-C levels and increased CVD risk.^57^ Commercial formulations of MCTs (as oils, powders or part of enteral nutrition formulations) are often purified to contain only MCTs, thus claiming greater health benefits, but these claims have not been substantiated with high quality studies.

MCTs differ fundamentally in digestion, absorption, and metabolism from long-chain triglycerides (LCTs) (that have 14+ carbons). After ingestion, MCTs are hydrolyzed by gastric and pancreatic lipases into medium-chain fatty acids, which are absorbed directly into the portal vein and transported to the liver bound to albumin. In the liver, medium-chain fatty acids bypass the need for carnitine transport and undergo rapid β-oxidation and ketogenesis.^58^ This contrasts with LCTs, which require emulsification by bile salts, packaging into chylomicrons, and transport via the lymphatic system to the tissues. Chylomicrons—triglyceride-rich lipoproteins that circulate in the bloodstream—are hydrolyzed by lipoprotein lipase (LPL), an enzyme anchored to the capillary endothelium in adipose tissue and muscle. LPL activity is essential for the clearance of LCTs.

### Evidence for the product

In conditions such as familial chylomicronemia syndrome (FCS), in which LPL function is absent or impaired, chylomicron clearance is severely compromised, leading to extreme hypertriglyceridemia and increased risk of acute pancreatitis. MCTs bypass this requirement entirely, providing a safe and essential source of calories in this context.^58^ Notably, coconut oil is not a true MCT oil for FCS because lauric acid (∼50% of coconut oil fat) behaves like a long-chain fatty acid requiring micellar solubilization with processing into chylomicrons rather than directly entering the portal circulation. Consequently, coconut oil should not be used as an MCT preparation for FCS. Beyond FCS, MCTs are also commonly used for ketogenic diets for refractory epilepsy, where they support ketosis and may reduce seizure frequency,^59^ malabsorption syndromes, such as pancreatic insufficiency or short bowel syndrome, and cachexia and metabolic disorders, where rapid hepatic oxidation can support energy needs.^60^

MCTs have been publicly promoted for weight loss. Small RCTs have shown short-term weight loss (0.5-1.4 kg over 4-12 weeks),^61^ increased energy expenditure (5-10% above baseline),^62^ or neutral or modestly favorable effects on high-density lipoprotein cholesterol and insulin sensitivity.^63^^,^^64^ More than a dozen RCTs have evaluated the effects of various MCT formulations, compared to other fats, on blood lipid levels. For example, one cross-over trial among 28 obese, insulin-resistant men found no plasma lipid effects of 20 g/day MCT oil vs 20 g/day PUFA-rich corn oil over 4 weeks.^65^ Another 12-week trial in 41 healthy adults using direct kinetic tracers found no effects on apolipoprotein B, a casual risk factor for CVD, comparing a coffee with butter and MCT oil to regular coffee.^66^ A meta-analysis of 13 RCTs of MCT oils concluded that MCTs have no significant effects on LDL-C, compared to other oils.^67^

Several RCTs have evaluated the effects of MCTs on body weight and composition. A meta-analysis of 13 trials using C8:0 and C10:0 MCTs in healthy adults identified modest but significant benefits for body weight (−0.51 kg), waist circumference (−1.46 cm), body fat (standard mean difference [SMD] −0.39), subcutaneous fat (SMD −0.46), and visceral fat (SMD −0.55), without effects on blood lipids.^61^ Another meta-analysis of 4 RCTs in adults with overweight or obesity found that pure MCTs produced modest weight loss (−1.62%), with significant reduction in blood triglycerides and no change in LDL-C.^68^ Overall, however, MCT studies have been typically short in duration, involved small sample sizes, and may not be generalizable to broader populations.

### Evidence against the product

There have been no long-term RCTs assessing CVD risk associated with MCT intake, and long-term health effects remain uncertain.

### Dietary guideline recommendations

Both the 2021 AHA Scientific Statement^17^ and 2025 DGAC report^4^ recommend limiting saturated fat and favor replacement with unsaturated plant oils for CVD health. However, MCT-rich oils were not specifically considered in these reports. Although the occasional MCT use may have minimal impact on cholesterol levels per se, long-term guidance prioritizes unsaturated fats to lower LDL-C and overall CVD risk.

### MCT oils: bottom line

MCTs have utility as a specialized therapeutic tool with established roles in conditions like FCS, malabsorption, and ketogenic therapy for epilepsy. However, their extrapolation to the general population is premature. Until high-quality trials are available, MCT supplementation should not be broadly recommended for cardiometabolic health. Finally, coconut oil is not an MCT and should not be used for FCS.

## Seafood

### Food description

Seafood is a source of high-quality protein, the marine omega-3 fatty acids eicosapentaenoic acid (EPA) and docosahexaenoic acid (DHA), and many micronutrients including vitamin D, B_12_, selenium, and zinc.^69^ The EPA and DHA content of seafood varies and generally reflects the fat content and diet of the species. Fish high in omega-3 fatty acids (so-called fatty or oily fish) include salmon, herring, sardines, mackerel, rainbow trout, and tuna. All salmon (farm raised and wild caught) are high in EPA and DHA. White or warm water fish generally contain less EPA and DHA due to lower tissue fat content.^70^

### Evidence for the product

Many studies have demonstrated cardiovascular and other health benefits of seafood, supported by at least some positive cardiovascular trials of fish oil supplements, resulting in dietary recommendations to consume at least 8 weekly ounces of a variety of seafood, especially oily or fatty fish.^4^ The DGAs concluded that there is strong evidence from prospective cohort studies and RCTs that eating patterns that include seafood are associated with a reduced risk of CVD.^71^, ^72^, ^73^ They include the AHA recommendation of 1 to 2 seafood meals per week to reduce risk of CVD, including congestive heart failure, coronary heart disease, ischemic stroke, and sudden cardiac death.^73^^,^^74^

### Evidence against the product

Although seafood consumption provides net health benefits in observational studies and trials, it can also be a source of organic pollutants, microplastics, organophosphates, and heavy metals, such as mercury in certain fish species.^75^ Risk can be minimized by following federal and local advisories and by the use of proper cooking techniques. The Environmental Protection Agency has issued guidance about the consumption of high mercury fish, that is, king mackerel, orange roughy, marlin, shark, swordfish, tilefish, and bigeye tuna, for women who are or may become pregnant.^76^ At levels typically consumed, mercury exposure has no known chronic harm in adults.

Questions have been raised about meeting the increasing global demand for seafood^77^ and doing this in an environmentally sustainable manner that optimizes the nutritional quality of seafood.^78^ The National Oceanic and Atmospheric Administration^79^ maintains a list of species that are threatened and endangered and can be avoided.

### Dietary guideline recommendations

Both the 2021 AHA^17^ and 2025 DGAC report^4^ recommend consuming seafood, especially fatty fish, at least 2 servings per week as part of a heart-healthy diet, due to its benefits for lowering cardiovascular risk through omega-3 fatty acids. Seafood is emphasized as a preferred protein source over red and processed meats for long-term cardiovascular and overall health.

### Seafood: bottom line

Seafood is a heart-healthy food due to inherent cardioprotective ingredients (eg, omega-3 fatty acids). Although certain species may contain harmful environmental contaminants, net health effects are positive, and guidance can be followed to minimize exposure. Overfishing and contaminants remain a significant concern, outside the purview of this manuscript.

## Alternative sweeteners

### Food description

Although the first artificial sweetener, saccharin, was isolated in 1879, alternative sweeteners gained particular prominence starting in the 1990s, with the increased focus on obesity and energy reduction.^80^ At present, 8 non-nutritive sweeteners have been approved by the FDA: 6 artificial/synthetic (aspartame, advantame, acesulfame potassium, neotame, saccharin, and sucralose) and 2 originally identified from natural sources (monk fruit extract and stevia).^81^ Sugar alcohols (eg, erythritol, mannitol, sorbitol, and xylitol) are other reduced-calorie alternatives, providing 0.2 to 2.6 calories per gram compared to sugar’s 4 calories per gram.^82^ Although initially considered to be inert sugar substitutes, accumulating evidence from large-scale cohort studies and mechanistic investigations suggests that alternative sweeteners may pose cardiovascular risks.

### Evidence for the product

#### Artificial sweeteners

Several studies have evaluated the health effects of replacing SSBs with drinks edulcorated with artificial or non-nutritive sweeteners. For example, a 2024 meta-analysis of RCTs found that replacing SSBs with low- or no-calorie sweetened beverages was associated with a mean body weight reduction of ≈1.06 kg, lower body mass index (BMI), lower percent body fat, and reduced intrahepatocellular lipid.^83^ A 2023 systematic review and meta-analysis of longer term substitution (≥6 months) studies of SSBs with noncaloric beverages (artificially sweetened or unsweetened beverages) showed a sustained BMI reduction of 0.31 kg/m^2^ (≈0.5-1 kg depending on age/height).^84^ These followed an earlier meta-analysis of RCTs reporting that the use of low-calorie sweeteners (instead of caloric sugars) modestly but significantly reduced body weight (∼−0.8 kg), BMI, fat mass, and waist circumference.^85^^,^^86^

#### Naturally occurring sweeteners

Currently, no research has investigated the impact of stevia or monk fruit on CVD, although some studies suggest potential positive effects on cardiovascular risk factors. For example, both compounds contain high levels of antioxidants, and thereby may reduce inflammation, oxidative stress, and ultimately, atherosclerosis development.^87^ RCTs suggest that stevia can improve blood pressure and blood sugar regulation,^88^ whereas animal studies suggest that monk fruit may improve lipid profiles.^87^ However, given that they are relatively new to the mass market, as more evidence emerges, some discordance may arise. In addition, sugar alcohols (carrying risks as denoted above) are often combined with stevia and monk fruit as bulking agents to better emulate sugar.^88^

### Evidence against the product

#### Artificial sweeteners

A *C*irculation 2019 analysis in women found consuming ≥2 servings/day of artificially sweetened beverages (ASBs) was associated with a 10% higher relative risk of all-cause mortality and a 15% higher relative risk of cardiovascular mortality, with a dose-dependent effect—up to 30% higher relative risk of mortality with ≥4 servings/day.^89^ In parallel, the NutriNet-Santé cohort demonstrated a 32% higher risk of cardiovascular events among high consumers of artificial sweeteners compared to nonconsumers.^90^ In the Women’s Health Observational Study, ≥2 ASB servings/day were associated with a 23% increased risk of stroke.^91^ Proposed underlying mechanisms include increased cardiovascular risk factors (hypertension and dyslipidemia), gut dysbiosis, compromised endothelial-dependent vasodilation, insulin resistance, and upregulation of inflammation.^92^^,^^93^

#### Sugar alcohols

Recent mechanistic evidence provides biological plausibility linking sugar alcohols, specifically erythritol and xylitol, to thrombotic risk. In human intervention trials, the ingestion of a single dose of erythritol or xylitol resulted in a significant enhancement of platelet aggregation, and flow cytometry demonstrated increased expression of activation markers.^94^ In addition, a recent in vitro study offered another mechanism via which sugar alcohols may increase CVD risk; it found that erythritol upregulated oxidative stress and downregulated nitric oxide production.^95^ Overall, elevated erythritol and xylitol levels have both been associated with increases in major adverse cardiovascular events, including myocardial infarction and stroke, up to 3-fold and 1.57-fold, respectively.^96^

#### Naturally occurring sweeteners

In a well-conducted RCT,^97^ stevia produced adverse microbiome changes over 2 weeks, with unclear repercussions.

### Dietary guideline recommendations

The 2021 AHA^17^ and 2025 DGAC report^4^ recommend limiting added sugars and note that sugar alcohols and naturally occurring sweeteners (eg, stevia and monk fruit) can be used occasionally as lower-calorie alternatives, but should not replace whole, minimally processed foods. Emphasis remains on reducing overall sweetness preference and prioritizing water, fruits, and unsweetened foods and beverages for cardiovascular and metabolic health.

### Alternative sweeteners: bottom line

From a public health perspective, some artificial sweeteners seem to carry more risk than has been previously thought. Although reverse causation can confound some of the interpretation of data, the totality of the evidence suggests potential for harm. Conversely, limited research suggests a benefit of ASB over SSBs in lowering the risk of coronary events.^98^^,^^99^ Overall, given the current evidence, we recommend consuming water, tea, and coffee^100^^,^^101^ and limiting or avoiding artificial sweeteners.

#### Summary

In *Trending Cardiovascular Nutritional Controversies 2026,* the goal has been to provide evidence in support of or to refute topics that have come under scrutiny, mostly by nonscientific outlets, despite the lack of justifiable clinical data. Available evidence (Table 2) provides key publications, brief summaries, and conclusions supporting the recommendations related to the cardiovascular health benefits of controversial foods and non-nutrient sources (Central Illustration). Available evidence supports cardiovascular benefits of seed oils and seafood, with care to avoid oil-based oxidation and seafood contaminants, and similar health effects of whole fat and low-fat dairy. Evidence to date also suggests minimizing or eliminating the use of beef tallow, UPF and artificial sweeteners, and sugar alcohols due to adverse cardiovascular effects. Continued areas of uncertainty include health effects of MCT oils and natural occurring sweeteners, where additional research is needed.

**Table 2:** Key References Behind Recommendation

Nutrition/Food Item	Key Publication(s) on the Topic First Author, Ref #	A Brief Summary of the Study	Key Conclusions
**Beef tallow**	1. Denke and Grundy^9^ 2. Lichtenstein et al^10^	1. Randomized cross over study of healthy men consuming 40% of energy derived from fatty acids. 2. Randomized cross over study of those with moderate hypercholesterolemia.	1. Mean LDL-C was significant higher after beef fat consumption. 2. Subjects consuming beef tallow at 20% of daily energy had significant elevations in LDL-C when compared to corn oil.
**Ultraprocessed foods**	1. Juul et al^20^ 2. Hall et al^23^ 3. Fardet^27^ 4. Srour et al^31^	1. NHANES analysis of standardized 24-h dietary recalls across survey cycles 2001-2002 through 2017-2018. 2. Randomized trial, subjects received ultra-processed diet or unprocessed diet for 2 weeks followed by an alternative diet for 2 weeks. 3. Study evaluating food processing and satiety index. 4. Observational study using over 100,000 adults with reported exposure to nitrites and nitrates via 24-hour dietary recall and risk of T2DM.	1. Average UPF intake in U.S. adults rose to 57% of daily calories, while consumption of minimally processed foods declined. 2. UPF diet led to increase energy intake, specifically carbohydrates and fat. There was also increased weight gain vs weight loss in the unprocessed diet group. 3. Foods with greater processing using the NOVA classification, found to have greater glycemic index with decrease satiety 4. Over a 7.3-year follow up, highest intake of dietary nitrites increased risk of incident diabetes.
**Full-fat dairy**	1. Guo et al^33^ 2. Alvarez-Bueno et al^34^ 3. Chen et al^39^	1. Meta-analysis of 29 prospective cohort studies. 2. Systematic review and meta-analysis of epidemiological studies evaluating associations of milk and dairy product consumption with T2DM. 3. Prospective observational study evaluating the association between dairy fat and CVD.	1. Neutral association between dairy products and CVD and all-cause mortality. 2. Total dairy consumption associated with lower risk of T2DM, specifically for yogurt and low-fat dairy. 3. Dairy fat was not significant related to total CVD, CHD or stroke. However, replacing dairy fat with PUFA or vegetable fat was associated with a reduce relative risk of CVD.
**Seed oils**: canola oil, soybean oil, omega-6 fatty acid acids, vegetable omega-3 fatty acid (alpha-linolenic acid)	1. Mozaffarian et al^16^ 2. Ghobadi et al^42^ 3. Atefi et al^46^ 4. Su et al^47^ 5. Marklund et al^49^	1. Meta-analysis of 8 RCTs evaluating the intake of PUFA vs saturated fats (n = 13,614). 2. Meta-analysis of 27 RCTs (n = 1,359). 3. RCT of 77 women with T2DM who were either given canola, sunflower, or olive oil. 4. Meta-analysis of 30 RCTs. 5. Meta-analysis of 30 prospective observational studies using biomarkers of fatty acid intake.	1. There was a 19% reduction (RR: 0.81; 95% CI: 0.70-0.95; *P* = 0.008) in CHD for each 5% increase in PUFA consumption. Consuming PUFA in place of saturated fatty acids reduced CHD events. 2. Canola oil consumption reduced total LDL-C (−6.4 mg/dL, 95% CI: −10.8 to −2); it had no impact on HDL-C, triglyceride or apoB levels. 3. Anthropometric data and inflammatory markers were obtained at baseline and 8 weeks after intervention. There was a significant reduction in hs-CRP levels in the canola and olive oil groups. 4. Increased dietary linoleic acid did not raise inflammatory markers 5. Higher levels of linoleic acid were associated with lower rates of total CVD, mortality and stroke.
**MCT Oils**	1. Mumme and Stonehouse^61^	1. Meta-analysis of RCTs comparing the effects of MCT to LCTs on weight loss and body composition in adults (n = 749).	1. Across 13 trials, MCTs showed a statistically significant reduction in body weight, total body fat, waist circumference, subcutaneous fat and visceral fat (*P* < 0.001). There were no differences in lipid profiles.
**Seafood**	1. Critselis et al^71^ 2. Ricci et al^72^ 3. O’Keefe et al^74^	1. Prospective cohort study (n = 2,020) examining association between seafood intake and long-term (up to 20 years) incident CVD in healthy adults. 2. Meta-analysis (n = 1,442,407) examining association between fish intake and CVD risk. 3. International prospective cohorts study (n = 183,291) evaluating omega-3 levels and incident stroke.	1. High seafood intake (>2 servings/week) was associated with 27% decreased 10-year CVD risk compared to lower intake (HR: 0.73; 95% CI: 0.55-0.98). 2. Two portions of fish consumed weekly reduced the risk of CVD outcomes 9%. 3. Incident ischemic stroke was reduced 18% and 14% at the highest vs lowest levels of eicosapentanoic acid (*P* < 0.0001) and docosahexanoic acid (*P* = 0.0001), respectively.
**Artificial sweeteners**: aspartame, advantame, acesulfame potassium, neotame, saccharin, sucralose; monk fruit extract, stevia	1. Malik et al^89^ 2. Chazelas et al^90^ 3. Ma et al^98^	1. Used multiple cohorts: Health Professional’s Follow-up study and Nurses’ Health Study (n = 3,415,564) to examine the association of AS beverages and risk of mortality. 2. Prospective study using the NutriNet-Sante cohort to assess the association of intake of AS on CVD risk (n = 103,388). 3. Prospective cohort study evaluating beverage consumption and mortality among those with T2DM.	1. After adjusting for dietary and lifestyle factors, consumption of sugar sweetened beverages was associated with a higher risk of both cancer and CVD mortality (*P* < 0.0001). There was a non-statistically significant rise in cancer and CVD mortality with AS beverages. 2. Total AS intake was found to increase CVD (HR: 1.09; 95% CI: 1.01-1.18; *P* = 0.03), with a higher stroke risk than non-consumers. Aspartame was associated with an increase in cerebrovascular events (1.17; 95% CI: 1.03-1.33; *P* = 0.02) and acesulfame potassium and sucralose were both associated with an increased risk of CHD. 3. There was increased mortality among those who consumed increased sugar sweetened and AS beverages.

apoB = apolipoprotein B; CHD = coronary heart disease; CVD = cardiovascular disease; HDL-C = high density lipoprotein-cholesterol; hs-CRP = high-sensitive C-reactive protein; LCT = long chain triglycerides; LDL-C = low density lipoprotein-cholesterol; MCT Oil = medium-chain triglyceride oils; NHANES = National Health and Nutrition Examination Survey; PUFA = polyunsaturated fatty acid; RCT = randomized controlled trial; T2DM = type II diabetes mellitus; UPF = ultraprocessed foods.

**Central Illustration:**
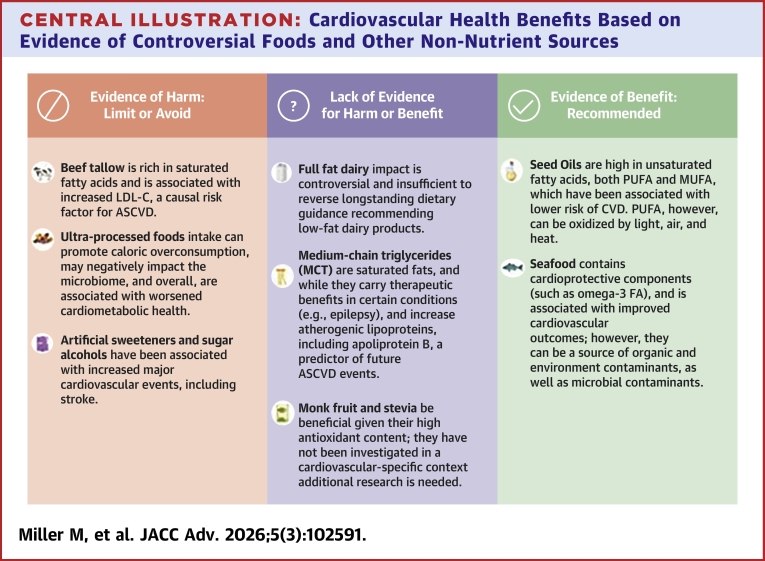
**Cardiovascular Health Benefits Based on Evidence of Controversial Foods and Other Non-Nutrient Sources** This illustration depicts health benefits of the controversial items discussed in this paper using the designations of “evidence of harm”, “lacking in evidence” and “evidence of benefit”. ASCVD = atherosclerotic cardiovascular disease; CVD = cardiovascular disease; LDL-C = low density lipoprotein-cholesterol; MUFA = monounsaturated fatty acid; PUFA = polyunsaturated fatty acid.

## Funding support and author disclosures

Dr Bhattacharya has received advisory/consulting fees from 10.13039/100004336Novartis, BetterLife Inc, and Parameter Health unrelated to current work. Dr Klodas is the founder of Step One Foods. Dr Mozaffarian is the member of the scientific advisory board of Brightseed, Calibrate, Filtricine, Instacart Health, January Inc., and WndrHLTH; has done scientific consulting from Amazon Health and Google Health; has equity in HumanCo; and has received chapter royalties from UpToDate. Dr Ostfeld has received research funding from the Purjes Foundation, the Greenbaum Foundation, and Beyond Meat, Inc; and holds options to purchase stock in Mesuron, Inc. Dr Petersen has received research grants from Cotton Incorporated, National Cattlemen’s Beef Association, Hass Avocado Board, American Pecan Council, American Egg Board, American Pistachio Growers, and McCormick Science Institute for unrelated work; and has received honoraria from the Soy Nutrition Institute Global, The Peanut Institute, and National Cattlemen’s Beef Association for unrelated work. Dr Ros has received research funding through his institution from the California Walnut Commission, Folsom, CA; personal fees and travel support for Advisory Board from Alexion; honoraria for lectures, presentations, and manuscript writing; travel support from Sociedad Española de Arteriosclerosis and Fundación Dieta Mediterránea, both from Spain; and travel support from European Society of Cardiology, Imperial College London, and the Wine Information Council. Dr Freeman has nothing to disclose related to this paper but does nonpromotional speaking or consulting for Boehringer-Ingelheim, Novartis, Agepha, Medtronic, Johnson & Johnson, Ionis, and United Therapeutics. All other authors have reported that they have no relationships relevant to the contents of this paper to disclose.

## Footnotes

∗ Fast-food restaurants that have switched to or use beef tallow (or beef shortening) include Steak 'n Shake, Outback Steakhouse, Popeyes, and Buffalo Wild Wings. Steak 'n Shake fully transitioned to 100% beef tallow by March 2025 for fries, onion rings, and chicken tenders. Outback Steakhouse has used beef fat for its fried foods, including the Bloomin' Onion, since 1988. Popeyes uses beef tallow for all its U.S. fried foods, and Buffalo Wild Wings uses beef shortening for its wings and other fried items.

∗∗ Several fast-food restaurants, including Steak 'n Shake, Popeyes, Outback Steakhouse, Buffalo Wild Wings, and Smashburger, have switched or plan to switch to cooking their products in beef tallow instead of seed oils. The move to beef tallow is driven by consumer interest in more traditional, natural ingredients and is supported by the Secretary of Health and Human Services, Robert F. Kennedy Jr, who has criticized seed oils.
